# Matrine regulates H_2_O_2_-induced oxidative stress through long non-coding RNA HOTAIR/miR-106b-5p axis via AKT and STAT3 pathways

**DOI:** 10.1042/BSR20192560

**Published:** 2020-05-26

**Authors:** Guanxue Xu, Wei Zhang, Zhenglong Wang, Man Chen, Bei Shi

**Affiliations:** Department of Cardiology, Affiliated Hospital of Zunyi Medical University, Zunyi 563000, China

**Keywords:** AKT pathway, HOTAIR, matrine, miR-106b-5p, oxidative stress myocardial cells

## Abstract

Matrine is a main active constituent of Chinese herb Sophora flavescens Ait (Kushen), which has shown various pharmacological effects, and has been reported to exhibit protective effects in heart failure. In the present study, the underlying mechanism of matrine was explored in H_2_O_2_-induced H9c2 cell line. It was confirmed that matrine could alleviate H_2_O_2_-induced injury in H9c2 cells. And the down-regulation of long non-coding RNA HOTAIR induced by H_2_O_2_ could be reversed by treating with matrine. Moreover, overexpression of HOTAIR promoted cell viability and superoxide dismutase (SOD) level, but inhibited cell apoptosis and lactate dehydrogenase (LDH) level. We found that miR-106b-5p was a target of HOTAIR and negatively regulated by HOTAIR. Moreover, up-regulation of miR-106b-5p restored the effects of HOTAIR overexpression on cell viability, apoptosis, and the levels of LDH and SOD. In addition, matrine protected H9c2 cells from H_2_O_2_-induced injury through HOTAIR/miR-106b-5p axis. Furthermore, we discovered that matrine exerted protective effects on H_2_O_2_-induced H9c2 cells through activating STAT3 and AKT pathway. In brief, matrine modulated H_2_O_2_-induced myocardial oxidative stress repair through HOTAIR/miR-106b-5p axis via AKT and STAT3 signaling pathway. Our study may provide a therapeutic target for the therapy of oxidative stress heart diseases.

## Introduction

Oxidative stress played an essential role in multiple diseases including heart failure [1,2], myocardial ischemia–reperfusion [3], and atherosclerosis [4,5]. H_2_O_2_, a reactive oxygen species with high reactivity, can promote the production of oxygen free radicals and induce myocardial apoptosis [6,7]. Hence, H_2_O_2_ is commonly used to establish cellular oxidative stress model *in vitro*. The present study aimed to clarify the regulatory mechanism of matrine in H_2_O_2_-induced oxidative damage model.

Accumulating evidence indicated that *Sophora flavescens* AIT was proved to be used to treat tumors, viral hepatitis, viral myocarditis, and arrhythmia [8,9]. Matrine, a major alkaloid isolated from *Sophora flavescens* AIT, played an utter role in ontogenesis. For example, matrine exerted suppressive effects on various tumors [10]. Moreover, matrine participated in the treatment of chronic hepatitis B [11]. Previous studies indicated that matrine repressed cell proliferation and invasion in breast cancer and induced cell apoptosis in gastric carcinoma [12,13]. Moreover, matrine regulated cell apoptosis to improve the function of heart failure and the arrhythmogenic effect caused by ouabain could be prevented by matrine [14,15]. The study investigated the role of matrine in H_2_O_2_-induced cardiomyocytes *in vitro*.

Long non-coding RNAs (lncRNAs) were longer than 200 bases with no function of translation. LncRNAs were proved to participate in the pathogenesis of various diseases [16]. Previous researches suggested that lncRNAs contributed to epigenetic regulation and served as a bridge between RNA and cancer [17,18]. Moreover, lncRNAs were demonstrated to exert regulatory effects on heart failure [19]. LncRNA HOX transcript antisense RNA (HOTAIR) was confirmed to act as a master regulator of chromatin dynamics and cancer [20]. More importantly, HOTAIR was closely related to heart diseases. For example, a previous study reported that HOTAIR was decreased in oxidative stimuli-induced H9c2 cells, and inhibition of HOTAIR could aggravate H9c2 cell injury induced by oxidative stress by suppressing MMP2 expression via targeting miR-125 [21]. Gao and colleagues found that HOTAIR was down-regulated and HOTAIR overexpression could attenuate hypoxia-triggered apoptosis of cardiomyocytes via regulating miR-1 [22]. Besides, accumulating evidence demonstrated that microRNAs (miRNAs) played crucial roles in oxidative stress related diseases [23]. For example, miR-106b-5p alleviated oxidative stress and ameliorated cerebral ischemia and reperfusion injury in rat model [24]. However, the underlying mechanism of HOTAIR and miR-106b-5p in heart diseases remains unclear.

H_2_O_2_ was often chose as a tool to model oxidative stress injury and H9c2 cells were frequently used to study oxidative stress induced cardiomyocyte apoptosis [25,26]. In the present study, we aimed to explore the effects of matrine on oxidative stress in cardiomyocytes. Moreover, we explored the role of HOTAIR and miR-106b-5p in oxidative stress induced cardiomyocytes. We confirmed that matrine regulated cell growth and apoptosis of H_2_O_2_-induced oxidative stressed H9c2 cells through HOTAIR/miR-106b-5p axis via AKT and STAT3 pathways.

## Materials and methods

### Cell culture and transient transfection

The rat cardiomyocytes H9c2 cells used in the present study were purchased from Be Na culture collection (Beijing, China). Dulbecco’s Modified Eagle Medium (DMEM, Hyclone, Logan, UT, U.S.A.) mixed with 10% fetal bovine serum (Thermo Fisher Scientific, Rockford, IL, U.S.A.) and 1× penicillin–streptomycin solution (Gibco, Carlsbad, CA, U.S.A.) was utilized to culture H9c2 cells at 37°C, 5% CO_2_. H9c2 cells were treated with H_2_O_2_ (100 µmol/l). For inhibitor experiments, wortmannin (100 nmol; Beyotime Institute of Biotechnology, Haimen, China) was a selective PI3K inhibitor and AG490 (50 µmol; Sigma-Aldrich, St Louis, MO, U.S.A.) was used as the JAK1/STAT3 inhibitor.

Overexpression vector of HOTAIR (HOTAIR) and the blank control (NC), small interference RNA targeting HOTAIR (si-HOTAIR) and its negative control (si-NC), miR-106b-5p mimics and the control (miR-NC) were synthesized by Sangon Biotech (Shanghai, China). Cells were transfected with the relative vectors using lipofectamine™3000 (Invitrogen, Carlsbad, CA, U.S.A.).

### CCK-8 assay

Cell Counting Kit-8 (Sigma-Aldrich, St. Louis, MO, U.S.A.) was used to determine cell viability. Cells were incubated for 48 h before adding WST-8 (Sigma-Aldrich) with concentration of 0.5 mg/ml and then H9c2 cells were incubated for another 2 h at 37°C. Finally, a microplate reader (Bio-Rad Laboratories, Philadelphia, PA, U.S.A.) was used to measure the optical density at 450 nm.

### Flow cytometry

Cell apoptosis was detected by Annexin V Apoptosis Detection Kit I (Solarbio, Beijing, China). After washing, H_2_O_2_-induced H9c2 cells were suspended using 100 µl binding buffer. Then, Annexin V and PI were added and incubated for 10 min at room temperature in dark. Finally, cell apoptosis was detected via flow cytometer (BD Biosciences, San Jose, CA, U.S.A.).

### Western blot assay

Total protein was collected using lysis buffer (Sangon Biotech). The concentration of protein solution was quantified with BCA Protein Assay Kit (Sangon Biotech). Proteins were separated by sodium dodecyl sulfate (SDS)-polyacrylamide gel electrophoresis. Then, the proteins were transferred onto PVDF membrane (Sangon Biotech). Subsequently, proteins were incubated with the special primary antibodies: B-cell lymphoma-2 (Bcl-2; catalog on ab196495, 1:1000), BCL2-Associated X (Bax, catalog no ab32503, 1:5000), cleaved-caspase3 (catalog no ab49822, 1:500), STAT3 (catalog no ab119352, 1:5000), phospho-STAT3 (catalog no ab76315, 1:2000) and GAPDH (catalog no ab9485, 1:2500) purchased from Abcam (Cambridge, MA, U.S.A.), and protein kinase B (AKT) (catalog no. SAB4500799), phospho-AKT (catalog no. SAB4503853) purchased from Sigma-Aldrich.

### Detection of lactate dehydrogenase (LDH) and superoxide dismutase (SOD)

The level of LDH and SOD was measured by lactate dehydrogenase assay kit and SOD activity assay kit (Jiancheng Bioengineering Institute, China) following the manufacturer’s instructions, respectively.

### Quantitative real-time polymerase chain reaction (qRT-PCR)

The total RNA was extracted by Trizol (Invitrogen) and reversed to cDNA by using SuperScript™ IV First-Strand Synthesis System (Thermo Fisher Scientific). Subsequently, reaction solution was mixed with template and AceQ Universal SYBR qPCR Master Mix (Vazyme, Nanjing, China) according to producer’s instruction. U6 and GAPDH were used as internal controls and the levels of special RNAs were calculated via 2^−ΔΔCt^ method. Primers used in the study were as follows: HOTAIR: (forward: 5′-CCAGCGCTAAGTCCTTCCAG-3′, reverse: 5′-TCTACGTTGGCGCTGACTTC-3′); miR-106b-5p: (forward: 5′-CAAGTACCCACAGTGCGGT-3′, reverse: 5′-CTCGCTTCGGCAGCACA-3′); U6: (forward: 5′-CGCTTCGGCAGCACATATACTA-3′, reverse: 5′-CGCTTCACGAATTTGCGTGTCA-3′); GAPDH: (forward: 5′-AATGGGCAGCCGTTAGGAAA-3′, reverse: 5′- TGAAGGGGTCATTGATGGCA-3′).

### Dual-luciferase reporter assay

Briefly, HOTAIR-wt or HOTAIR-mut and miR-106b-5p or NC were co-transfected into H9c2 cells according to manufacturer’s instruction. Subsequently, dual-luciferase assay kit (Promega) was used to examine the luciferase activity on the base of protocol and the signals were collected by Varioskan Flash (Thermo Fisher Scientific).

### RNA immunoprecipitation (RIP) assay

The Imprint® RNA immunoprecipitation kit (Sigma-Aldrich) was used to verify the relationship between HOTAIR and miR-106b-5p. First, H9c2 cells were lysed by RIP-buffer. Subsequently, the lysed solution was incubated with magnetic beads coated AGO2 (Argonaute-2, Abcam) or IgG (Abcam) antibody. Then, the residuum was washed and the RNA was harvested. qRT-PCR was performed to detect the enrichment of HOTAIR and miR-106-5p.

### RNA pull-down

In the assay, bio-labeled probe of miR-106b-5p (Bio-miR-106b-5p) and blank control (Bio-miR-NC) were synthesized by Sangon (Shanghai, China). Then Bio-miR-106b-5p or Bio-miR-NC was transfected into H9c2 cells. Subsequently, cells were lysed and incubated with Streptavidin-Dyna beads overnight at 4°C along with RNA separation. Finally, the enrichment of HOTAIR was identified by qRT-PCR.

### Statistical analysis

The data were exhibited as mean ± standard deviation of three independent assays. Two-group and multiple-group comparisons were calculated via Student’s *t*-test and one-way analysis of variance, respectively. *P* less than 0.05 was considered as significant difference.

## Results

### Matrine increased cell viability and blocked apoptosis in H_2_O_2_-induced H9c2 cell model

**Figure 1:**
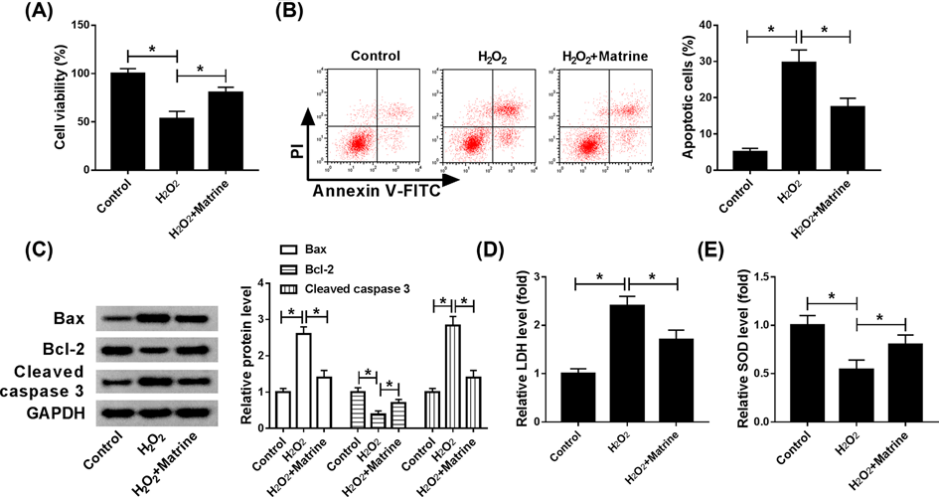
Matrine increased cell viability and blocked apoptosis in H_2_O_2_-induced H9c2 cell model (**A–E**) H_2_O_2_ was used to induce myocardial damage, and matrine was applied to retard H_2_O_2_-induced oxidative stress in H9c2 cells. (**A**) CCK-8 assay was used to detect cell viability after added matrine into H9c2 cells. (**B**) Flow cytometry was conducted to measure cell apoptosis. (**C**) Levels of apoptosis-related protein, Bax, Bcl-2 and Cleave-caspase 3 were detected by Western blot. (**D** and **E**) Levels of LDH and SOD in H_2_O_2_-induced H9c2 cells were analyzed by Elisa; *P<0.05.

In order to investigate the effect of matrine on H_2_O_2_-induced cardiomyocytes, 100 µmol/l H_2_O_2_ was used to induce H9c2 cells in the present study. About 50 µmol/l matrine increased the viability of oxidative stressed cardiomyocytes (Figure 1A), while apoptosis was notably decreased by treating with matrine compared with H_2_O_2_ group (Figure 1B). The change of apoptosis-related proteins of Bax, Bcl-2 and cleaved-caspase 3 also verified the conclusion that matrine inhibited cell apoptosis *in vitro* (Figure 1C). Moreover, the results showed that the level of LDH was significantly repressed, while SOD was promoted by matrine in comparison with the H_2_O_2_ group (Figure 1D,E). In brief, matrine could alleviate oxidative stress damage in H_2_O_2_-induced H9c2 cells.

### HOTAIR was enhanced and miR-106b-5p was repressed by matrine in H_2_O_2_-induced H9c2 cells

**Figure 2:**
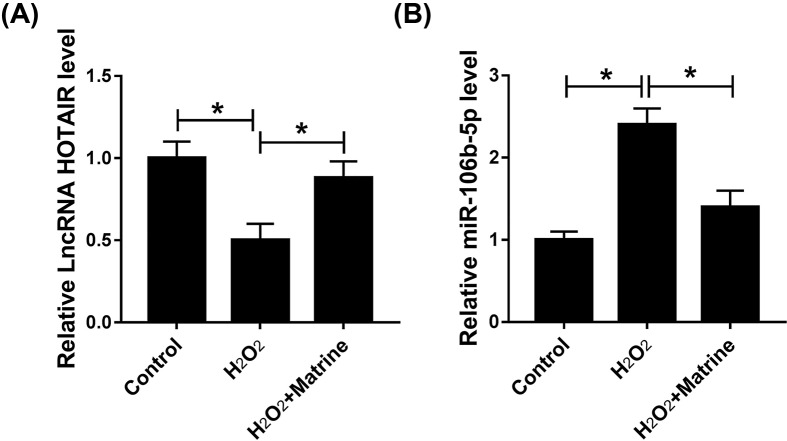
HOTAIR was enhanced and miR-106b-5p was repressed by matrine in H_2_O_2_-induced H9c2 cell model (**A** and **B**) The levels of HOTAIR and miR-106b-5p were measured by qRT-PCR; *P<0.05.

To further research the potential molecular mechanism of matrine in oxidative stress, H_2_O_2_-induced H9c2 cells were treated with matrine and cultured for 48 h, then the levels of HOTAIR and miR-106b-5p were measured by qRT-PCR. Results showed that the level of HOTAIR was enhanced (Figure 2A), while miR-106b-5p was dramatically hindered by matrine in oxidative stressed cardiomyocytes (Figure 2B). These data suggested that matrine might regulate oxidative stress in H9c2 cells through HOTAIR and miR-106b-5p.

### Up-regulation of HOTAIR induced cell viability, hindered apoptosis and decreased the oxidative stress damage in H_2_O_2_-induced H9c2 cells

**Figure 3:**
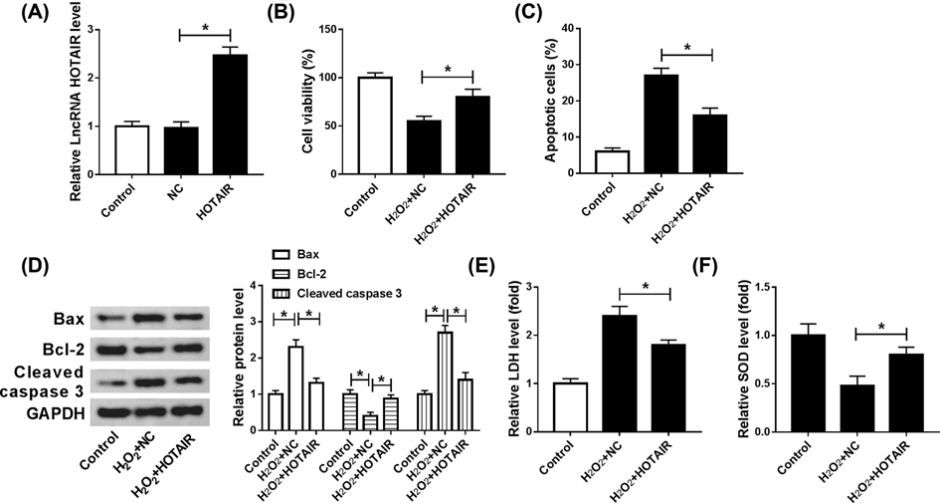
Up-regulation of HOTAIR induced cell viability, hindered apoptosis and decreased oxidative stress damage in H_2_O_2_-induced cardiomyocytes (**A–F**) The level of HOTAIR was determined by qRT-PCR (**A**) after transfected with HOTAIR or NC in H9c2 cells. (B–F) H_2_O_2_-induced cardiomyocytes were transfected with HOTAIR or NC, respectively. (**B**) Cell viability was assessed by CCK-8. (**C**) Flow cytometry was conducted to evaluate cell apoptosis *in vitro*. (**D**) Apoptosis-relative proteins of Bcl-2, Bax and Cleaved-caspase 3 were determined by Western blot. ELISA was carried out to examine the level of LDH and SOD; *P<0.05.

To further explore the role of HOTAIR in oxidative stressed cardiomyocytes *in vitro*, overexpression vector of HOTAIR (HOTAIR) and blank control (NC) were transfected into H_2_O_2_-induced cardiomyocytes. The up-regulation efficiency of HOTAIR was verified and showed in Figure 3A. Then, CCK-8 assay was carried to determine cell viability and results showed that up-regulation of HOTAIR remarkably enhanced cell viability in oxidative stressed cardiomyocytes (Figure 3B). Moreover, cell apoptosis was notably decreased in H_2_O_2_-induced H9c2 cells transfected with HOTAIR (Figure 3C). Besides, cell apoptosis was also verified by the inhibition of Bax and cleaved caspase 3 expression and promotion of Bcl-2 expression (Figure 3D). The decreased level of LDH and increased level of SOD were found in oxidative stressed cardiomyocytes treated with matrine (Figure 3E,F). In short, up-regulation of HOTAIR eased oxidative stress damage in H_2_O_2_-induced cardiomyocytes.

### MiR-106b-5p was a direct target of HOTAIR

**Figure 4:**
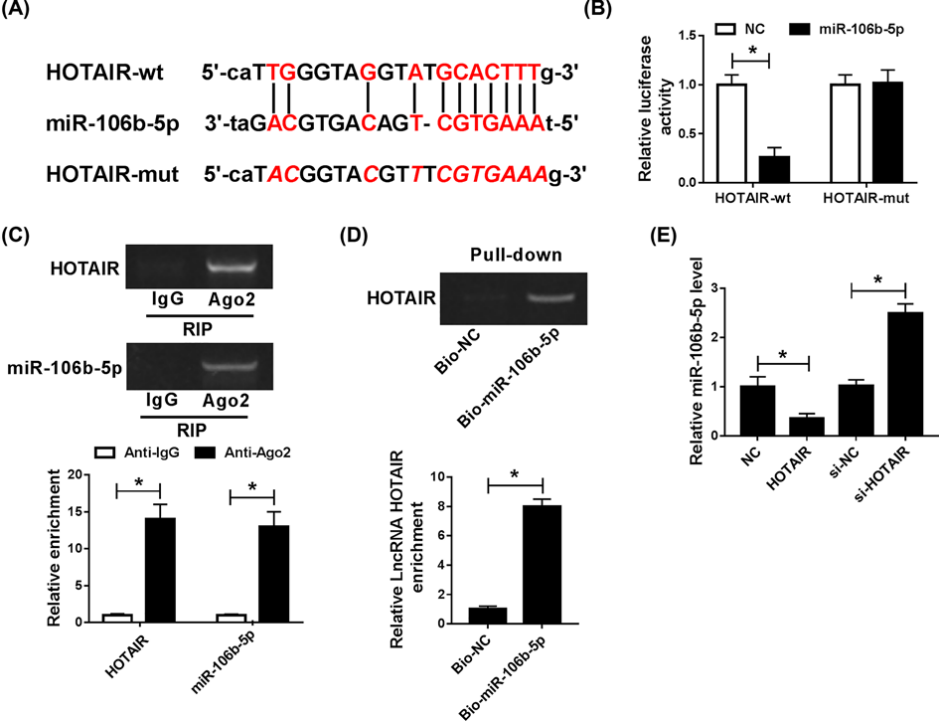
MiR-106b-5p was a target gene of HOTAIR and was regulated by HOTAIR (**A**) The binding sites of HOTAIR and miR-106b-5p were predicted via Starbase v2.0 (**B–D**) and dual-luciferase reporter and RIP, as well as RNA pull-down assays were performed to verify the relationship between miR-106b-5p and HOTAIR. The results of agarose electrophoresis of the PCR products were showed. (**E**) The level of miR-106b-5p was identified by qRT-PCR after knockdown of HOTAIR *in vitro*;*P<0.05.

The binding sites between HOTAIR and miR-106b-5p were predicted by Starbase v2.0 (Figure 4A). The relationship between HOTAIR and miR-106b-5p was verified by dual-luciferase reporter assay, RIP and RNA pull-down assays. The repression luciferase activity in wild-type group clarified that miR-106b-5p was directly targeted by HOTAIR (Figure 4B). In addition, RIP and RNA pull-down assays were both clarified the interaction between HOTAIR and miR-106b-5p (Figure 4C,D). These results revealed that miR-106b-5p was directly targeted by HOTAIR. Additionally, the expression of miR-0106b-5p was down-regulated by HOTAIR up-regulation, while miR-106b-5p expression was increased by down-regulating HOTAIR (Figure 4E). In conclusion, miR-106b-5p was a direct target of HOTAIR and negatively regulated by HOTAIR.

### Overexpression of miR-106b-5p reversed the effects of HOTAIR up-regulation on cell viability, apoptosis and oxidative stress damage in H_2_O_2_-induced H9c2 cells

**Figure 5:**
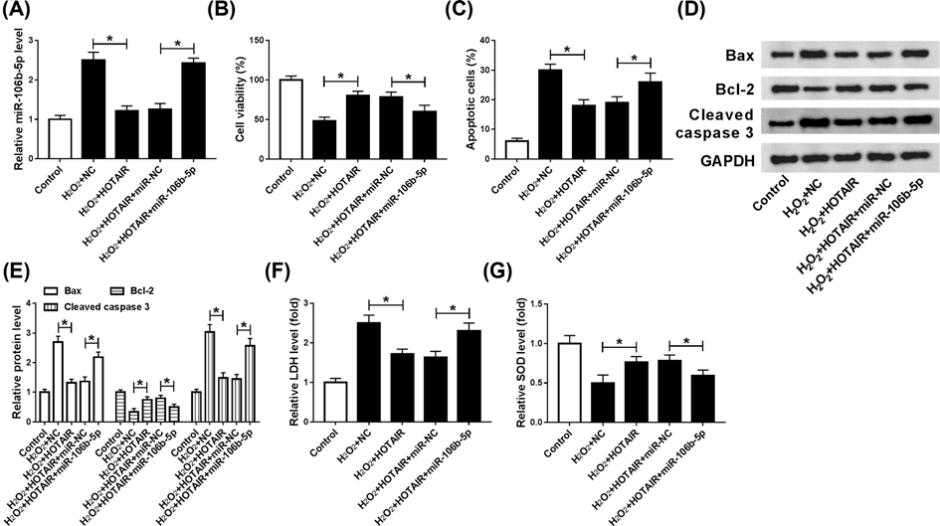
Overexpression of HOTAIR heightened cell viability, curbed apoptosis and oxidative damage, while these effects were reversed by miR-106b-5p in H_2_O_2_-induced cardiomyocytes (**A–G**) NC, HOTAIR, HOTAIR + miR-NC or HOTAIR + miR-106b-5p into H9c2 cells were transfected into H9c2 cells induced by H_2_O_2_. (**A**) The level of miR-106-5p was examined by qRT-PCR. (**B**) The cell viability was measured by CCK-8. (**C**) Flow cytometry was employed to detect cell apoptosis *in vitro*. (**D**) Western blot assay was aimed to examine apoptosis-relative proteins expression. The level of LDH and SOD were identified by ELISA; *P<0.05.

Then, the underlying mechanism of HOTAIR and miR-106b-5p in oxidative stressed H9c2 cells was investigated. First, H_2_O_2_-induced H9c2 cells were transfected with NC, HOTAIR, HOTAIR + miR-NC and HOTAIR + miR-106b-5p. The level of miR-106b-5p was strikingly decreased by overexpression of HOTAIR, while rescued by miR-106-5p mimics (Figure 5A). The promotion effect of HOTAIR up-regulation on cell viability was regained by overexpression of miR-106b-5p (Figure 5B). Additionally, overexpression of HOTAIR suppressed cell apoptosis, while the suppressive effect was reversed by miR-106b-5p mimics (Figure 5C). Furthermore, the effects of HOTAIR on the expression of Bax, Bcl-2 and Cleaved caspase 3 were abolished by miR-106b-5p mimics (Figure 5D,E). These results indicated that the suppressive effect of HOTAIR on cell apoptosis was attenuated by miR-106b-5p overexpression in H_2_O_2_-stimulated oxidative stressed cell model. Besides, up-regulation of miR-106b-5p also reversed the effects of HOTAIR on the levels of LDH and SOD in H_2_O_2_-induced H9c2 cells (Figure 5F,G). In brief, miR-106b-5p overexpression restored the effects of HOTAIR overexpression on cell viability, apoptosis and oxidative stress damage in oxidative stressed H9c2 cells.

### The effects of matrine on cell viability, apoptosis and oxidative stress damage in H_2_O_2_-induced H9c2 cells were abolished by HOTAIR down-regulation or miR-106b-5p up-regulation

**Figure 6:**
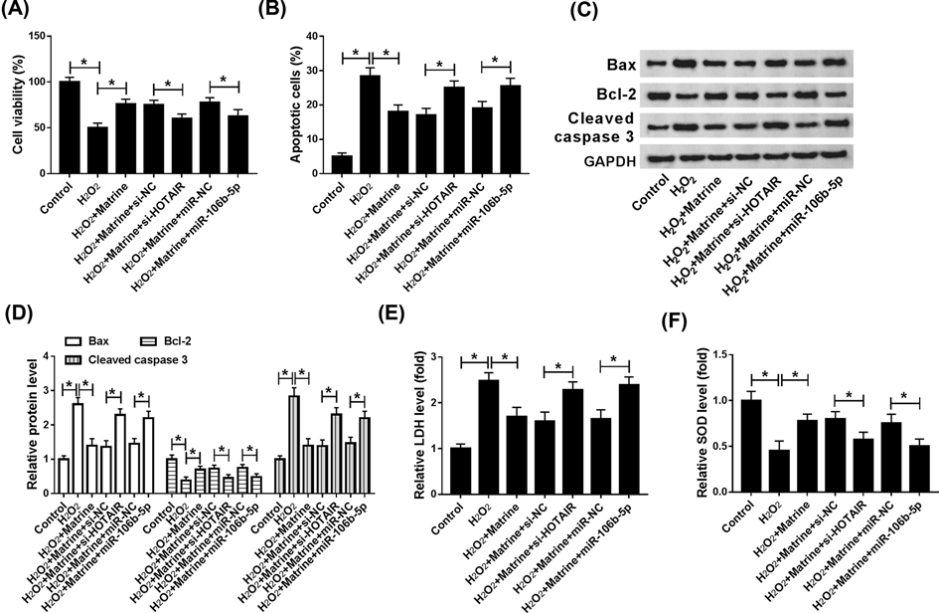
The effects of matrine on H_2_O_2_-induced cell viability, apoptosis and oxidative stress damage were abolished by HOTAIR down-regulation or miR-106b-5p up-regulation (**A–F**) Knockdown vector of HOTAIR (si-HOTAIR) and negative control (si-NC) were constructed, and then si-NC, si-HOTAIR, miR-NC or miR-106b-5p was transfected with into H_2_O_2_-induced H9c2 cells that was added matrine. (**A**) Cell viability was determined by qRT-PCR. (**B**) Cell apoptosis was examined by using flow cytometry. (**C** and **D**) The expression of relative-proteins Bax, Bcl-2 and Cleave-caspase 3 was measured via Western blot to verified cell apoptosis *in vitro*. (**E** and **F**) The level of LDH and SOD was analyzed by ELISA assay. *P<0.05.

To further investigate the association among HOTAIR, matrine and miR-106b-5p in oxidative stressed H9c2 cells. H_2_O_2_-induced H9c2 cells treated with matrine were transfected si-NC, si-HOTAIR, miR-NC or miR-106b-5p. The results showed that the promotion effect of matrine on cell viability and inhibitory effect on apoptosis were reversed in si-HOTAIR group or miR-106b-5p group (Figure 6A,B). In addition, the effect of matrine on cell apoptosis was also verified by detection of Bax, Bcl-2 and Cleaved caspase 3 expression in H_2_O_2_-induced H9c2 cells (Figure 6C,D). Furthermore, the levels of LDH and SOD modified by matrine were also restrained by knockdown of HOTAIR or up-regulation of miR-106b-5p (Figure 6E,F). Overall, HOTAIR down-regulation or miR-106b-5p overexpression recovered the effects of matrine on cell viability, apoptosis and the expression of LDH and SOD in H_2_O_2_-induced oxidative stressed H9c2 cells.

### Matrine regulated cell viability, apoptosis and oxidative stress through HOTAIR/miR-106b-5p axis via AKT and STAT3 signaling pathways

**Figure 7:**
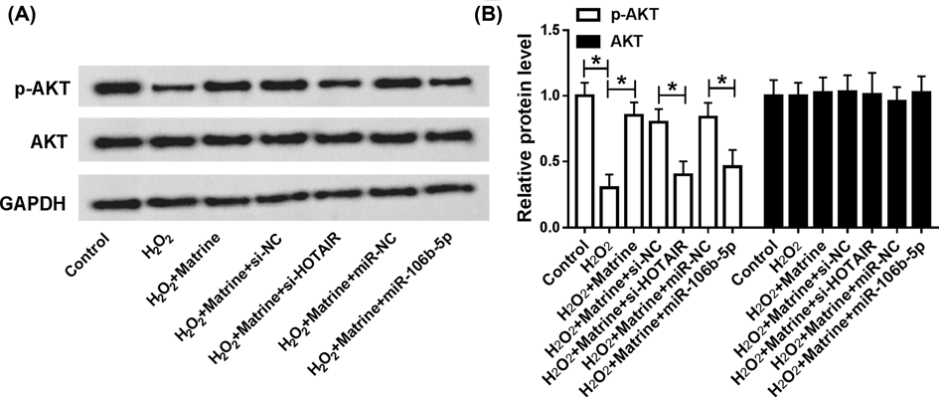
Matrine regulated cell viability, apoptosis and oxidative stress damage through HOTAIR/miR-106b-5p axis via AKT signaling pathway (**A** and **B**) The levels of p-AKT and AKT were detected and quantified after transfection with matrine, matrine + si-NC, matrine + si-HOTAIR, matrine + miR-NC or matrine + miR-106b-5p into H_2_O_2_-induced H9c2 cells; *P<0.05.

**Figure 8:**
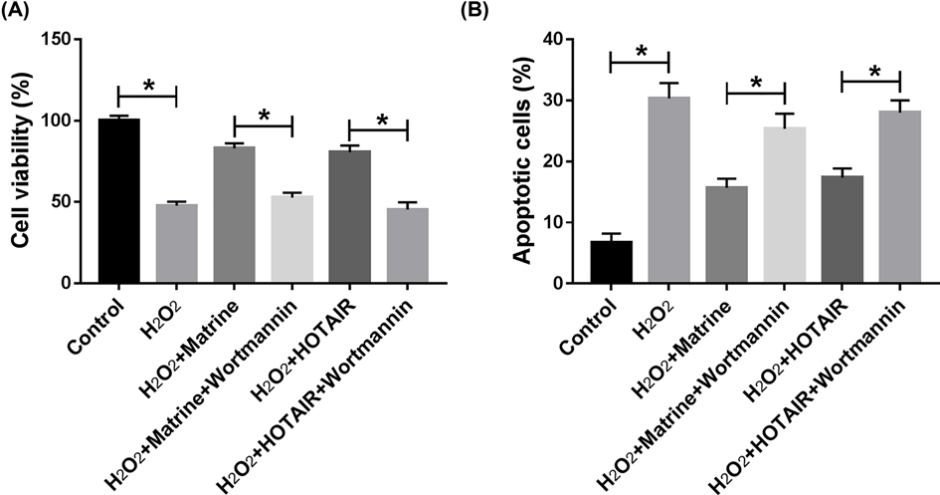
Wortmannin reversed the effects of matrine on cell proliferation and apoptosis in H_2_O_2_-induced H9c2 cells (**A**) Cell viability was detected by CCK-8 assay. (**B**) Cell apoptosis was measured by flow cytometry. *P<0.05.

**Figure 9:**
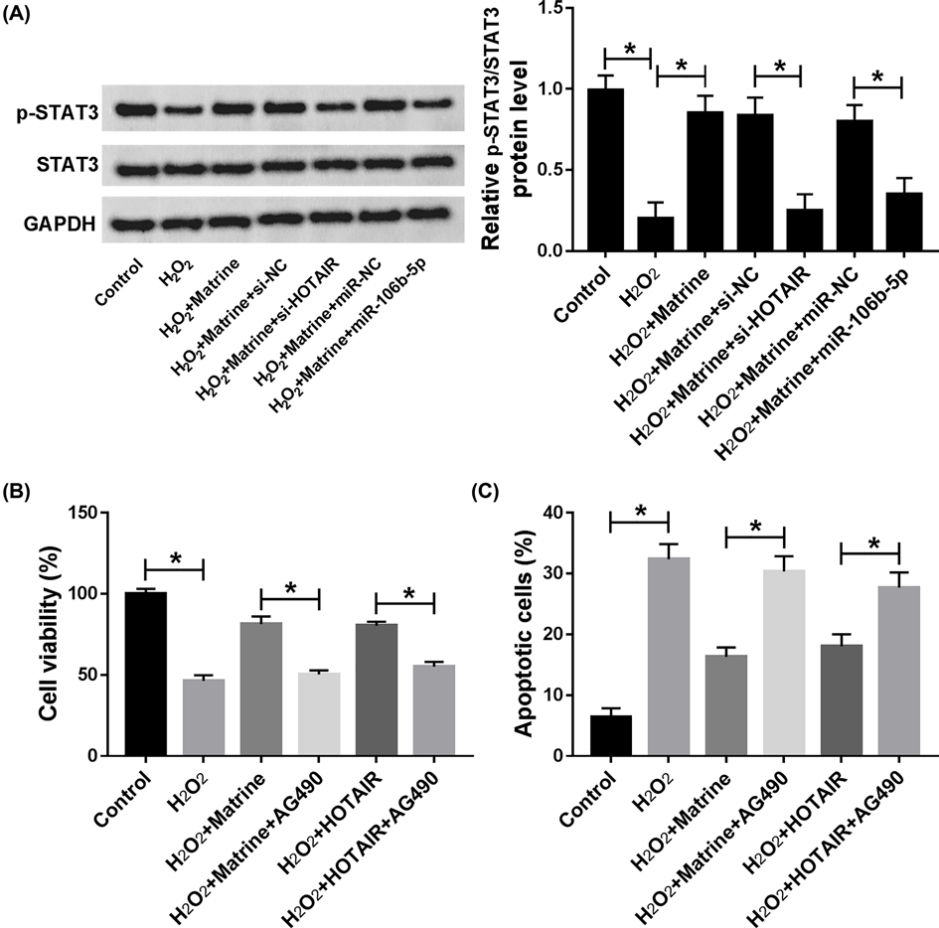
Matrine regulated cell viability, apoptosis and oxidative damage through HOTAIR/miR-106b-5p axis via STAT3 signaling pathway (**A**) The levels of p-STAT3 and STAT3 were determined by Western blot. (**B**) Cell viability was detected by CCK-8 assay. (**C**) Cell apoptosis was measured by flow cytometry; *P<0.05.

In order to research the special mechanism of matrine, the expression of AKT, p-AKT, STAT3 and p-STAT3 in oxidative stressed cardiomyocytes was measured and quantified in H_2_O_2_-induced H9c2 cells. The results proved that matrine promoted the expression of p-AKT, which was reversed by knockdown of HOTAIR or miR-106b-5p overexpression in H_2_O_2_-induced cardiomyocytes (Figure 7A,B). Besides, our results indicated that the promotion effect on cell viability and inhibition effect on cell apoptosis of matrine or HOTAIR were abolished by AKT3 inhibitor Wortmannin, suggesting that matrine exerted protective effects on H_2_O_2_-induced H9c2 cells through regulating HOTAIR-mediated activation of Akt pathway (Figure 8A,B). As shown in Figure 9A, we found that pSTAT3/STAT3 ratio was increased in H_2_O_2_-induced H9c2 cells, which was reversed by HOTAIR deletion or miR-106b-5p overexpression. Our data also showed that matrine-mediated increase in cell viability and decrease in cell apoptosis in H_2_O_2_-induced H9c2 cells were hindered by STAT3 inhibitor AG490 (Figure 9B,C). In conclusion, the regulatory mechanism of HOTAIR/miR-106b-5p axis modulated the role of matrine in oxidative stressed H9c2 cells at least partial via AKT and STAT3 signaling pathways.

## Discussion

Accumulating evidence indicated that apoptosis is closely related to several cardiovascular diseases pathological process including heart failure, cardiac ischemia, reperfusion injury, myocardial infarction and atrial fibrillation [27–30]. Oxidative stress was proved as a vital regulator factor in the progression and initiation of human diseases including cardiovascular diseases. It has been demonstrated that antioxidants exerted effects on preventing oxidative stress-mediated cardiovascular dysfunction [31]. In our study, we found that matrine could enhanced cell viability and inhibited cell apoptosis, as well as alleviated oxidative stress in H_2_O_2_-induced H9c2 cells, which was consistent with the previous study from Hu and colleagues [32]. Therefore, investigating the potential mechanism of matrine on oxidative stress induced cardiomyocytes growth and apoptosis was important for the effective therapy of oxidative stress-related diseases.

Previous researches have confirmed that HOTAIR could regulate oxidative stress damage and play a crucial role in regulating cells growth and apoptosis in oxidative stress stimuli cardiomyocytes [33]. These previous studies indicated that HOTAIR exerted vital effects on heart function. Our study revealed that H_2_O_2_ decreased HOTAIR expression, while matrine could up-regulate the expression of HOTAIR in H_2_O_2_-induced H9c2 cells. Moreover, HOTAIR up-regulation could alleviate the H_2_O_2_-induced cell apoptosis and oxidative stress and accelerated the proliferation of H9c2 cells, suggesting that HOTAIR might be involved in the protective effects of matrine on H_2_O_2_-induced H9c2 cells.

Starbase v2.0 was utilized to predict the binding sites of HOTAIR and microRNAs (miRNAs), the result showed that HOTAIR had complementary fragments with miR-106b-5p. MiRNAs were involved in multiple diseases including but not limiting cancers [34] and oxidative stress [35]. For instance, miR-223 facilitated cell invasion in gastric cancer [36], and miR-144 regulated oxidative stress in sickle cell disease [37]. Moreover, miR-106b-5p regulated the development of breast cancer [38], non-small cell lung cancer [39] and glioma [40]. Besides, miR-106b-5p was also proved to be closely associated with cell apoptosis and oxidative stress [24]. Our data showed that miR-106b-5p was a direct target of HOTAIR and negatively regulated by HOTAIR. Besides, miR-106b-5p overexpression could reverse the protective effects of HOTAIR on H_2_O_2_-induced oxidative stress damage. These results suggested that matrine exhibited protective effects on H_2_O_2_-induced H9c2 cells through HOTAIR/miR-106b-5p axis.

Previous study has demonstrated that HOTAIR exerted promotion effect on cell growth and inhibition effect on cell apoptosis in endometrial carcinoma by suppressing PTEN to activate PI3K/AKT pathway [41]. In another research, Qi et al*.* reported that the activation of PI3K/AKT pathway-mediated by HOTAIR exhibited improvement effects on diabetic cardiomyopathy through facilitating the viability of cardiomyocytes [42]. These studies suggested that HOTAIR played a vital role in endometrial carcinoma and diabetic cardiomyopathy by activating PI3K/AKT pathway. Furthermore, previous studies revealed that H_2_O_2_-induced apoptosis of cardiomyocytes and the inhibition of PI3K/AKT pathway were observed in H_2_O_2_-induced cardiomyocytes [43]. Consistent with the previous studies, our data showed that the PI3K/AKT and STAT3 pathway were inhibited by HOTAIR down-regulation or miR-106b-5p up-regulation, and matrine increased the expression of HOTAIR to alleviate H_2_O_2_-stimulated oxidative stress in H9c2 cells through sponging miR-106b-5p to activate AKT and STAT3 pathways.

In short, the study discovered that matrine alleviated H_2_O_2_-induced oxidative stress in cardiomyocytes. Moreover, HOTAIR directly targeted miR-106b-5p to regulate the effect of matrine on oxidative stress damage. Further experiments verified that matrine alleviated oxidative stress through HOTAIR/miR-106b-5p axis via AKT and STAT3 signaling pathways. However, there were some limitations in the present research, the rat H9c2 cells not human cardiomyocytes were used in the present study. Thus, the results might be not necessarily applicable on human cardiomyocytes. Besides, the *in vivo* experiments using an animal model of myocardial injury might be helpful to explore the results in physiological and pathological processes.

## Conclusion

**Figure 10:**
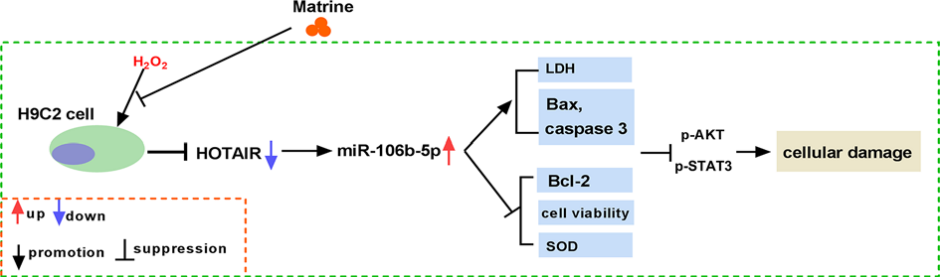
The regulatory mechanism of matrine in H_2_O_2_-induced oxidative stress in cardiomyocytes Matrine regulated HOTAIR/miR-106b-5p axis, thereby activating AKT and STAT3 pathway to H_2_O_2_-induced alleviate oxidative stress in cardiomyocytes.

In the study, matrine was proved to ease H_2_O_2_-induced oxidative stress in H9c2 cells. And matrine could regulate the levels of HOTAIR and miR-106b-5p. Furthermore, miR-106-5p was a target gene of HOTAIR. Overexpression of HOTAIR increased cell viability, blocked apoptosis and alleviated oxidative stress. Moreover, miR-106b-5p reversed the effects of HOTAIR overexpression on cell viability, apoptosis, LDH and SOD levels in H_2_O_2_-induced H9c2 cells. Besides, down-regulation of HOTAIR or up-regulation of miR-106b-5p restored the effects of marine on repairing oxidative stress injury in H9c2 cells. In conclusion, matrine alleviated cardiac myocytes oxidative stress damage through HOTAIR/miR-106b-5p network via AKT and STAT3 pathway in H_2_O_2_-induced cell model (Figure 10). The present study provided a novel insight into the effects and underlying mechanism of matrine on oxidative stress in cardiomyocytes.

## Abbreviations

Bax: BCL2-Associated X
Bcl-2: B-cell lymphoma-2
CCK-8: cell counting kit-8
HOTAIR: HOX transcript antisense RNA
LDH: lactate dehydrogenase
miR-106b-5p: microRNA-106-5p
qRT-PCR: quantitative real-time polymerase chain reaction
RIP: RNA immunoprecipitation
SOD: superoxide dismutase

## References

[1] Tsutsui H., Kinugawa S. and Matsushima S. (2011) Oxidative stress and heart failure. Am. J. Physiol. Heart Circ. Physiol. 301, H2181–H2190 10.1152/ajpheart.00554.2011 21949114

[2] Giordano F.J. (2005) Oxygen, oxidative stress, hypoxia, and heart failure. J. Clin. Invest. 115, 500–508 10.1172/JCI200524408 15765131 PMC1052012

[3] Santos-Gallego C.G., Vahl T.P., Goliasch G., Picatoste B., Arias T. et al. (2016) Sphingosine-1-Phosphate Receptor Agonist Fingolimod Increases Myocardial Salvage and Decreases Adverse Postinfarction Left Ventricular Remodeling in a Porcine Model of Ischemia/Reperfusion. Circulation 133, 954–966 10.1161/CIRCULATIONAHA.115.012427 26826180

[4] Santos-Gallego C.G., Picatoste B. and Badimon J.J. (2014) Pathophysiology of acute coronary syndrome. Curr. Atheroscler. Rep. 16, 401 10.1007/s11883-014-0401-9 24504549

[5] Santos-Gallego C.G., Bayon J. and Badimon J.J. (2010) Thrombi of different pathologies: implications for diagnosis and treatment. Curr. Treat. Options Cardiovasc. Med. 12, 274–291 10.1007/s11936-010-0075-8 20842548

[6] Sies H. (2014) Role of metabolic H2O2 generation redox signaling and oxidative stress. J. Biol. Chem. 289, 8735–8741 10.1074/jbc.R113.544635 24515117 PMC3979367

[7] Dudek E.J., Shang F. and Taylor A. (2001) H2O2-mediated oxidative stress activates NF-κB in lens epithelial cells. Free Radic. Biol. Med. 31, 651–658 10.1016/S0891-5849(01)00634-7 11522450

[8] Ma L., Wen S., Zhan Y., He Y., Liu X. and Jiang J. (2008) Anticancer effects of the Chinese medicine matrine on murine hepatocellular carcinoma cells. Planta Med. 74, 245–251 10.1055/s-2008-1034304 18283616

[9] Jin J.H., Kim J.S., Kang S.S., Son K.H., Chang H.W. and Kim H.P. (2010) Anti-inflammatory and anti-arthritic activity of total flavonoids of the roots of Sophora flavescens. J. Ethnopharmacol. 127, 589–595 10.1016/j.jep.2009.12.020 20034551

[10] Zhang M. and Huang J. (2004) Recent research progress of anti-tumor mechnism matrine. Zhongguo Zhong Yao Za Zhi 29, 115–118 15719674

[11] Long Y., Lin X., Zeng K. and Zhang L. (2004) Efficacy of intramuscular matrine in the treatment of chronic hepatitis B. Hepatobiliary Pancreat. Dis. Int. 3, 69–72 14969841

[12] Yu P., Liu Q., Liu K., Yagasaki K., Wu E. and Zhang G. (2009) Matrine suppresses breast cancer cell proliferation and invasion via VEGF-Akt-NF-κB signaling. Cytotechnology 59, 219–229 10.1007/s10616-009-9225-9 19760125 PMC2774574

[13] Dai Z.-j., Gao J., Ji Z.-z., Wang X.-j., Ren H.-t., Liu X.-x. et al. (2009) Matrine induces apoptosis in gastric carcinoma cells via alteration of Fas/FasL and activation of caspase-3. J. Ethnopharmacol. 123, 91–96 10.1016/j.jep.2009.02.022 19429345

[14] Yu J., Yang S., Wang X. and Gan R. (2014) Matrine improved the function of heart failure in rats via inhibiting apoptosis and blocking β3-adrenoreceptor/endothelial nitric oxide synthase pathway. Mol. Med. Rep. 10, 3199–3204 10.3892/mmr.2014.2642 25322941

[15] Zhou Y., Wu Y., Deng L., Chen L., Zhao D., Lv L. et al. (2014) The alkaloid matrine of the root of Sophora flavescens prevents arrhythmogenic effect of ouabain. Phytomedicine 21, 931–935 10.1016/j.phymed.2014.02.008 24680622

[16] Chen G., Wang Z., Wang D., Qiu C., Liu M., Chen X. et al. (2012) LncRNADisease: a database for long-non-coding RNA-associated diseases. Nucleic Acids Res. 41, D983–D986 10.1093/nar/gks1099 23175614 PMC3531173

[17] Mercer T.R. and Mattick J.S. (2013) Structure and function of long noncoding RNAs in epigenetic regulation. Nat. Struct. Mol. Biol. 20, 300 10.1038/nsmb.2480 23463315

[18] Yang G., Lu X. and Yuan L. (2014) LncRNA: A link between RNA and cancer. Biochim. Biophys. Acta 1839, 1097–1109 10.1016/j.bbagrm.2014.08.012 25159663

[19] Papait R., Kunderfranco P., Stirparo G.G., Latronico M.V. and Condorelli G. (2013) Long noncoding RNA: a new player of heart failure? J. Cardiovasc. Transl. Res. 6, 876–883 10.1007/s12265-013-9488-6 23835777 PMC3838575

[20] Arunoday B. and Mandal S.S. (2015) LncRNA HOTAIR: A master regulator of chromatin dynamics and cancer. Biochim. Biophys. Acta 1856, 151–164 26208723 10.1016/j.bbcan.2015.07.001 PMC4544839

[21] Li L., Zhang M., Chen W., Wang R., Ye Z., Wang Y. et al. (2018) LncRNA-HOTAIR inhibition aggravates oxidative stress-induced H9c2 cells injury through suppression of MMP2 by miR-125. Acta Biochim. Biophys. Sin. 50, 996–1006 10.1093/abbs/gmy102 30239560

[22] Gao L., Liu Y., Guo S., Yao R., Wu L., Xiao L. et al. (2017) Circulating Long Noncoding RNA HOTAIR is an Essential Mediator of Acute Myocardial Infarction. Cell. Physiol. Biochem. 44, 1497–1508 29258067 10.1159/000485588

[23] Ke J., Bian X., Liu H., Li B. and Huo R. (2019) Edaravone reduces oxidative stress and intestinal cell apoptosis after burn through up-regulating miR-320 expression. Mol. Med. 25, 54 10.1186/s10020-019-0122-1 31829167 PMC6907153

[24] Li P., Shen M., Gao F., Wu J., Zhang J., Teng F. et al. (2017) An antagomir to microRNA-106b-5p ameliorates cerebral ischemia and reperfusion injury in rats via inhibiting apoptosis and oxidative stress. Mol. Neurobiol. 54, 2901–2921 10.1007/s12035-016-9842-1 27023223

[25] Wu H., Gao H., Gao S., Lei Z., Dai L., Wang X. et al. (2019) A Chinese 4-herb formula, Yiqi-Huoxue granule, alleviates H2O2-induced apoptosis by upregulating uncoupling protein 2 in H9c2 cells. Phytomedicine 53, 171–181 10.1016/j.phymed.2018.09.031 30668396

[26] Liu F., Zhang H., Zhang Z., Lu Y. and Lu X. (2019) MiR-208a aggravates HO-induced cardiomyocyte injury by targeting APC. Eur. J. Pharmacol. 864, 1–9 10.1016/j.ejphar.2019.01.004 31545986

[27] Al-Gubory K.H., Fowler P.A. and Garrel C. (2010) The roles of cellular reactive oxygen species, oxidative stress and antioxidants in pregnancy outcomes. Int. J. Biochem. Cell Biol. 42, 1634–1650 10.1016/j.biocel.2010.06.001 20601089

[28] Hauton D. (2012) Hypoxia in early pregnancy induces cardiac dysfunction in adult offspring of Rattus norvegicus, a non-hypoxia-adapted species. Comp. Biochem. Physiol. A Mol. Integr. Physiol. 163, 278–285 10.1016/j.cbpa.2012.07.020 22892476

[29] Nanduri J., Makarenko V., Reddy V.D., Yuan G., Pawar A., Wang N. et al. (2012) Epigenetic regulation of hypoxic sensing disrupts cardiorespiratory homeostasis. Proc. Natl Acad. Sci. U.S.A. 109, 2515–2520 10.1073/pnas.1120600109 22232674 PMC3289330

[30] Patterson A.J. and Zhang L. (2010) Hypoxia and fetal heart development. Curr. Mol. Med. 10, 653–666 10.2174/156652410792630643 20712587 PMC3075953

[31] Kane A.D., Herrera E.A., Camm E.J. and Giussani D.A. (2013) Vitamin C prevents intrauterine programming of in vivo cardiovascular dysfunction in the rat. Circ. J. 77, 2604–2611 10.1253/circj.CJ-13-0311 23856654

[32] Hu C., Zhang X., Wei W., Zhang N. et al. (2019) viaMatrine attenuates oxidative stress and cardiomyocyte apoptosis in doxorubicin-induced cardiotoxicity maintaining AMPK/UCP2 pathway. Acta Pharm. Sin. B. 9, 690–701 10.1016/j.apsb.2019.03.003 31384530 PMC6664099

[33] Li L., Zhang M., Chen W., Wang R., Ye Z., Wang Y. et al. (2018) LncRNA-HOTAIR inhibition aggravates oxidative stress-induced H9c2 cells injury through suppression of MMP2 by miR-125. Acta Biochim. Biophys. Sin. (Shanghai) 50, 996–1006 10.1093/abbs/gmy102 30239560

[34] Wang K., Chen M. and Wu W. (2017) Analysis of microRNA (miRNA) expression profiles reveals 11 key biomarkers associated with non-small cell lung cancer. World J. Surg. Oncol. 15, 175 10.1186/s12957-017-1244-y 28927412 PMC5606074

[35] Leung A.K. and Sharp P.A. (2010) MicroRNA functions in stress responses. Mol. Cell 40, 205–215 10.1016/j.molcel.2010.09.027 20965416 PMC2996264

[36] Li X., Zhang Y., Zhang H., Liu X., Gong T., Li M. et al. (2011) miRNA-223 Promotes Gastric Cancer Invasion and Metastasis by Targeting Tumor Suppressor EPB41L3. Mol. Cancer Res. 9, 824–833 10.1158/1541-7786.MCR-10-0529 21628394

[37] Sangokoya C., Telen M.J. and Chi J.-T. (2010) microRNA miR-144 modulates oxidative stress tolerance and associates with anemia severity in sickle cell disease. Blood 116, 4338–4348 10.1182/blood-2009-04-214817 20709907 PMC2993631

[38] Li N., Liu Y., Miao Y., Zhao L., Zhou H. and Jia L. (2016) MicroRNA‐106b targets FUT6 to promote cell migration, invasion, and proliferation in human breast cancer. IUBMB Life 68, 764–775 10.1002/iub.1541 27519168

[39] Wei K., Pan C., Yao G., Liu B., Ma T., Xia Y. et al. (2017) MiR-106b-5p promotes proliferation and inhibits apoptosis by regulating BTG3 in non-small cell lung cancer. Cell. Physiol. Biochem. 44, 1545–1558 10.1159/000485650 29197876

[40] Liu F., Gong J., Huang W., Wang Z., Wang M., Yang J. et al. (2014) MicroRNA-106b-5p boosts glioma tumorigensis by targeting multiple tumor suppressor genes. Oncogene 33, 4813 10.1038/onc.2013.428 24166509

[41] Zhang X.H., Hu P., Xie Y.Q. et al. (2019) Long Noncoding RNA HOTAIR Promotes Endometrial Carcinoma Cell Proliferation by Binding to PTEN via the Activating Phosphatidylinositol 3-Kinase/Akt Signaling Pathway. Mol. Cell. Biol. 39, 10.1128/MCB.00251-19 PMC6851348 31527078

[42] Qi K. and Zhong J. (2018) LncRNA HOTAIR improves diabetic cardiomyopathy by increasing viability of cardiomyocytes through activation of the PI3K/Akt pathway. Exp. Ther. Med. 16, 4817–4823 30542437 10.3892/etm.2018.6755 PMC6257662

[43] Li Y., Xia J., Jiang N., Xian Y. et al. (2018) Corin protects HO-induced apoptosis through PI3K/AKT and NF-κB pathway in cardiomyocytes. Biomed. Pharmacother. 97, 594–599 10.1016/j.biopha.2017.10.090 29101802

